# Potential of Gold Nanoparticles for Noninvasive Imaging and Therapy for Vascular Inflammation

**DOI:** 10.1007/s11307-021-01654-5

**Published:** 2021-09-27

**Authors:** Hisanori Kosuge, Maki Nakamura, Ayako Oyane, Kazuko Tajiri, Nobuyuki Murakoshi, Satoshi Sakai, Akira Sato, Atsushi Taninaka, Taishiro Chikamori, Hidemi Shigekawa, Kazutaka Aonuma

**Affiliations:** 1 Department of Cardiology, Tokyo Medical University, 6-7-1 Nishishinjuku, Shinjuku-ku, 160-0023 Tokyo, Japan; 2 Nanomaterials Research Institute, National Institute of Advanced Industrial Science and Technology (AIST), Central 5, 1-1-1 Higashi, Tsukuba, Ibaraki 305-8565 Japan; 3 Department of Cardiology, Faculty of Medicine, University of Tsukuba, 1-1-1 Tennodai, Tsukuba, Ibaraki 305-8575 Japan; 4 Faculty of Pure and Applied Sciences, University of Tsukuba, 1-1-1 Tennodai, Ibaraki 305-8573 Tsukuba, Japan

**Keywords:** Gold nanoparticle, Inflammation, Computed tomography, Macrophage

## Abstract

**Purpose:**

Macrophages contribute to the progression of vascular inflammation, making them useful targets for imaging and treatment of vascular diseases. Gold nanoparticles (GNPs) are useful as computed tomography (CT) contrast agents and light absorbers in photothermal therapy. In this study, we aimed to assess the viability of macrophages incubated with GNPs after near-infrared (NIR) laser light exposure and to evaluate the utility of intravenously injected GNPs for *in vivo* imaging of vascular inflammation in mice using micro-CT.

**Procedures:**

Mouse macrophage cells (RAW 264.7) were incubated with GNPs and assessed for GNP cellular uptake and cell viability before and after exposure to NIR laser light. For *in vivo* imaging, macrophage-rich atherosclerotic lesions were induced by carotid ligation in hyperlipidemic and diabetic FVB mice (*n* = 9). Abdominal aortic aneurysms (AAAs) were created by angiotensin II infusion in ApoE-deficient mice (*n* = 9). These mice were scanned with a micro-CT imaging system before and after the intravenous injection of GNPs.

**Results:**

The CT attenuation values of macrophages incubated with GNPs were significantly higher than those of cells incubated without GNPs (*p* < 0.04). Macrophages incubated with and without GNPs showed similar viability. The viability of macrophages incubated with GNPs (100 μg/ml or 200 μg/ml) was decreased by high-intensity NIR laser exposure but not by low-intensity NIR laser exposure. *In vivo* CT images showed higher CT attenuation values in diseased carotid arteries than in non-diseased contralateral arteries, although the difference was not statistically significant. The CT attenuation values of the perivascular area in AAAs of mice injected with GNPs were significantly higher than those of mice without injection (*p* = 0.0001).

**Conclusions:**

Macrophages with GNPs had reduced viability upon NIR laser exposure. GNPs intravenously injected into mice accumulated in sites of vascular inflammation, allowing detection of carotid atherosclerosis and AAAs in CT imaging. Thus, GNPs have potential as multifunctional biologically compatible particles for the detection and therapy of vascular inflammation.

## Introduction

Atherosclerosis is widely accepted as a chronic, systemic vascular inflammatory disorder and remains the main cause of mortality in industrialized and developing nations [1]. Abdominal aortic aneurysm (AAA) is a common and lethal degenerative disease. AAA expansion is associated with the infiltration of inflammatory cells and degradation of the extracellular matrix in the arterial wall [2]. In vascular inflammation, macrophages play a key role in plaque formation, aneurysm progression, and rupture. Therefore, macrophages can be a useful target for the imaging and treatment of vascular diseases [3–5].

Gold nanoparticles (GNPs) can be used as contrast agents for X-ray computed tomography (CT) imaging of tumors and blood pools [6, 7]. CT imaging has demonstrated enhanced accumulation of GNPs conjugated with antibodies or peptides in targeted organs [8, 9]. Micro-CT imaging showed the uptake of GNPs into murine macrophages after co-incubation. In addition, GNPs were able to detect soft tissue inflammation in a murine hind leg edema model [10]. GNPs including gold nanorods, gold nanoshells, and gold nanocages can be used as light absorbers for photothermal ablation which convert photon energy to heat and induce hyperthermia upon exposure to laser light [11–13].

Thermal ablation therapies using laser, microwave, radiofrequency, and high-intensity focused ultrasound have been developed for the clinical treatment of cancers [14–17]. An important issue in these therapies is insufficient targetability; thermal ablation can damage healthy cells surrounding cancer cells. To overcome this challenge, previous studies have reported that thermal ablation therapy using a near-infrared (NIR) laser or an alternating magnetic field was effective for the treatment of cancer in mice injected with different types of nanoparticles [18, 19]. We have reported the effect of photothermal ablation of vascular macrophages using single-walled carbon nanotubes in a murine atherosclerotic model [20].

In this study, we evaluated the hypothesis that NIR laser exposure could reduce the viability of macrophages with GNPs and intravenously injected GNPs could image vascular inflammation in mice with micro-CT.

## Materials and Methods

### Cell Culture

Mouse macrophage cells (RAW 264.7) were cultured in Dulbecco’s modified Eagle’s medium (Sigma-Aldrich Co. LLC., USA) supplemented with 10% fetal bovine serum (Thermo Fisher Scientific Inc., USA) and 1% streptomycin/penicillin (Thermo Fisher Scientific Inc.) at 37 °C in a 5% CO_2_ atmosphere.

### *In Vitro *Uptake

GNPs (AuroVist™ 15 nm) were purchased from Nanoprobes, Inc. (USA). Macrophages were incubated with GNPs (0, 50, 100, and 200 μg/ml) at 37 °C under 5% CO_2_ for 24 h. After washing in phosphate-buffered saline (Thermo Fisher Scientific Inc.), the macrophages were collected and scanned with a micro-CT imaging system (LaTheta LCT-100A, Aloka Co., Japan). The CT attenuation values were calculated using an image-analysis software on a dedicated workstation (Ziostation2; Ziosoft, Inc., Japan).

### Cell Viability

Macrophages (1 × 10^5^/well) were seeded in 96-well plates and incubated at 37 °C under 5% CO_2_ for 24 h. Then, GNPs were added to the wells at different concentrations (0, 50, 100, and 200 μg/ml), and the macrophages were incubated for another 24 h. Next, 10 μl of a Cell Counting Kit-8 (CCK-8) solution (Dojindo Laboratories, Japan) was added to each well and the plates were incubated at 37 °C under 5% CO_2_ for 2 h. For each concentration group, wells without adding the CCK-8 solution were prepared as the corresponding negative control. The absorbance was measured at 450 nm in triplicate using a microplate reader (Varioskan LUX Multimode Microplate Reader, Thermo Fisher Scientific Inc.). The absorbance of corresponding negative controls was subtracted from the measured absorbance of each concentration group to compensate for the effect of GNPs. The results were expressed as relative values to cells without GNPs, which were set as 100% [21].

### *In Vitro *NIR Laser Exposure

Macrophages were incubated with GNPs (0, 100, and 200 μg/ml) at 37 °C under 5% CO_2_ for 24 h. Ten million cells with or without GNPs were exposed to NIR laser light (Mira-HP, COHERENT, Inc. USA, wavelength: 830 nm, pulse width: 200 fs, irradiation diameter: 1 cm^2^) for 0, 10, and 20 min at 178, 196, 400, and 437 mW. Macrophages with and without laser exposure were cultured at 37 °C under 5% CO_2_ for 24 h to evaluate their viability. The absorbance at 450 nm was measured in triplicate using CCK-8, as described above. The absorbance measured for the macrophages with laser exposure was normalized to that of the macrophages without laser exposure.

### *In Vivo *CT

All animal procedures were approved by the Institutional Animal Experiment Committee of the University of Tsukuba. All animals were anesthetized with ketamine and xylazine for surgical and imaging procedures and were allowed free access to food and water.

For carotid ligation, 8-week-old male FVB mice (*n* = 9) were fed a high-fat diet. After 4 weeks on the diet, diabetes was induced by five daily intraperitoneal injections of streptozotocin (STZ 40 mg/kg, Sigma-Aldrich Co. LLC.). Two weeks after the STZ injection, the left common carotid artery was ligated below the bifurcation to develop macrophage-rich neointimal proliferation. The non-ligated right common carotid artery served as the control [4]. Two weeks after the ligation, mice were injected with GNPs (5 FVB mice: 10 mg/mouse, 4 FVB mice: 20 mg/mouse) via the tail vein and scanned with a micro-CT imaging system at 24 and 48 h.

AAAs were induced in male apolipoprotein E-deficient mice, 14—24 weeks of age, by continuous angiotensin II infusion via subcutaneously implanted osmotic mini-pumps [22]. Two weeks later, transabdominal 40-MHz B-mode ultrasound imaging (Vevo 2100, VisualSonics Inc., Toronto, Canada) was performed to assess the aortic diameter. Mice with suprarenal aortic dissection (*n* = 9) were studied. Five mice were injected with GNPs (10 mg/mouse) via the tail vein and scanned with a micro-CT imaging system at 24 and 48 h. Four mice without injection were also scanned at 24 and 48 h. After *in vivo* imaging, the aortas were surgically exposed and *ex vivo* imaging was performed using a micro-CT imaging system.

### Statistical Analyses

All data are expressed as the mean ± standard error of the mean (SEM). The Kruskal–Wallis test was used to compare *in vitro* CT attenuation values and cell viability between macrophages with and without GNPs, followed by post hoc testing using the Mann–Whitney *U* test with Bonferroni adjustment. Two-way repeated measure analysis of variance was used to compare serial *in vivo* CT attenuation values between ligated left and non-ligated right common carotid arteries and between AAAs injected with GNPs and those without injection. The Wilcoxon signed-rank test was used to compare the ratio of CT attenuation values in the carotid arteries and AAAs between 24 and 48 h after injection of GNPs. The Mann–Whitney *U* test was used to compare cell viability between macrophages with and without GNPs after *in vitro* laser exposure and *ex vivo* CT attenuation values between AAAs injected with GNPs and those without injection. *P* < 0.05 was considered statistically significant.

## Results

### *In Vitro *Uptake and Viability

**Fig. 1:**
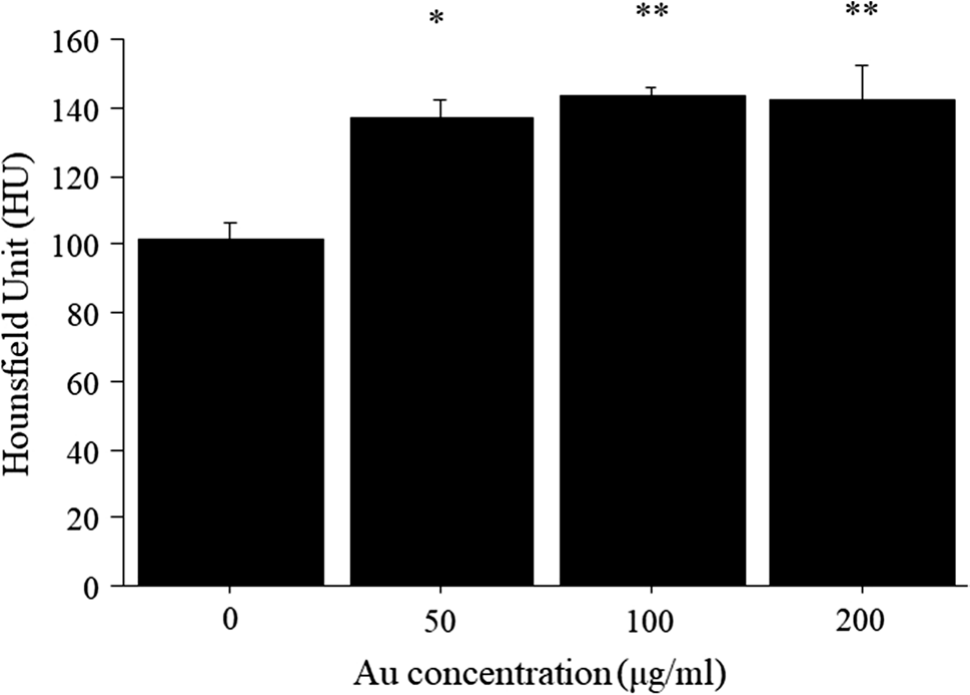
*In vitro* CT attenuation values of macrophages incubated with and without GNPs. The CT attenuation values of macrophages incubated with GNPs were significantly higher than those of macrophages without GNPs. *p < 0.03 *vs*. macrophages without GNPs, **p < 0.04 *vs*. macrophages without GNPs.

**Fig. 2:**
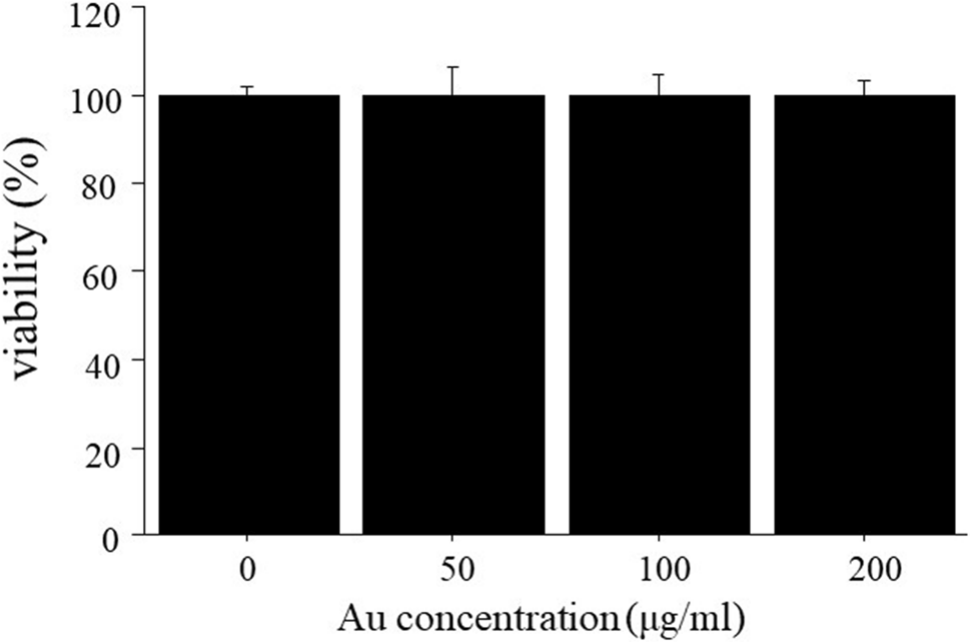
Cell viability by CCK-8 assay. Macrophages incubated with GNPs at a dose of 50, 100, or 200 μg/ml showed a similar viability as those without GNPs.

The CT attenuation values of macrophages incubated with GNPs at doses of 50, 100, and 200 μg/ml were significantly higher than those of cells without GNPs (Fig. 1). The viability of macrophages incubated with GNPs (50, 100, and 200 μg/ml) did not decrease compared to that of macrophages without GNPs (Fig. 2).

### *In Vitro *Laser Exposure

**Fig. 3:**
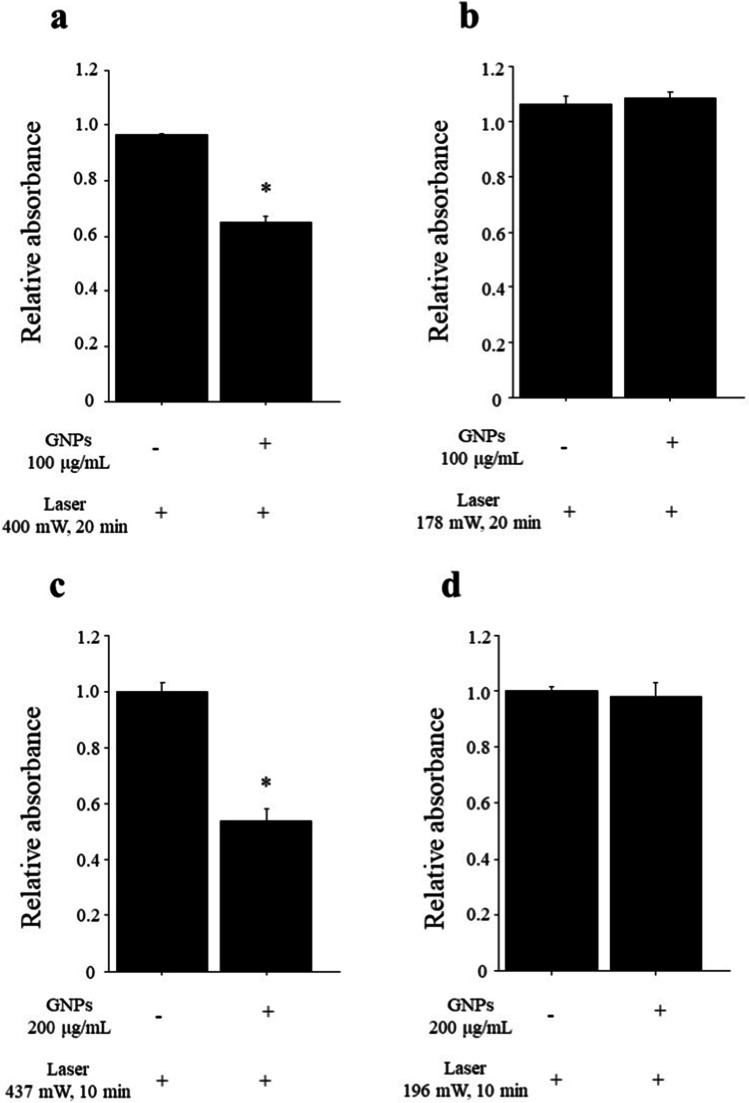
*In vitro* NIR laser exposure. **a**, **b** NIR laser exposure in macrophages incubated with and without GNPs (100 μg/ml). **a** Viability of macrophages irradiated with high-intensity NIR laser (400 mW) was significantly decreased by preincubation with GNPs. *p < 0.05. **b** Viability of macrophages irradiated with low-intensity NIR laser (178 mW) was not decreased by preincubation with GNPs. **c**, **d** NIR laser exposure in macrophages with and without GNPs (200 μg/ml). **c** Viability of macrophages irradiated with high-intensity NIR laser (437 mW) was significantly decreased by preincubation with GNPs. *p < 0.05. **d** Viability of macrophages irradiated with low-intensity NIR laser (196 mW) was not decreased by preincubation with GNPs.

High-intensity NIR laser light exposure significantly reduced the viability of macrophages treated with GNPs (100 μg/ml or 200 μg/ml) compared to macrophages without GNPs (100 μg/ml: 0.650 ± 0.018 *vs*. 0.962 ± 0.005, *p* < 0.05, Fig. 3a; 200 μg/ml: 0.539 ± 0.041 *vs*. 0.999 ± 0.030, *p* < 0.05, Fig. 3c). However, the viability of macrophages incubated with GNPs (100 μg/ml or 200 μg/ml) was not reduced by low-intensity NIR laser light exposure (100 μg/ml: 1.061 ± 0.034 *vs*. 1.088 ± 0.018, *p* = 0.8273, Fig. 3b; 200 μg/ml: 1.000 ± 0.018 *vs*. 0.981 ± 0.052, *p* = 0.8273, Fig. 3d).

### *In Vivo *CT Imaging

**Fig. 4:**
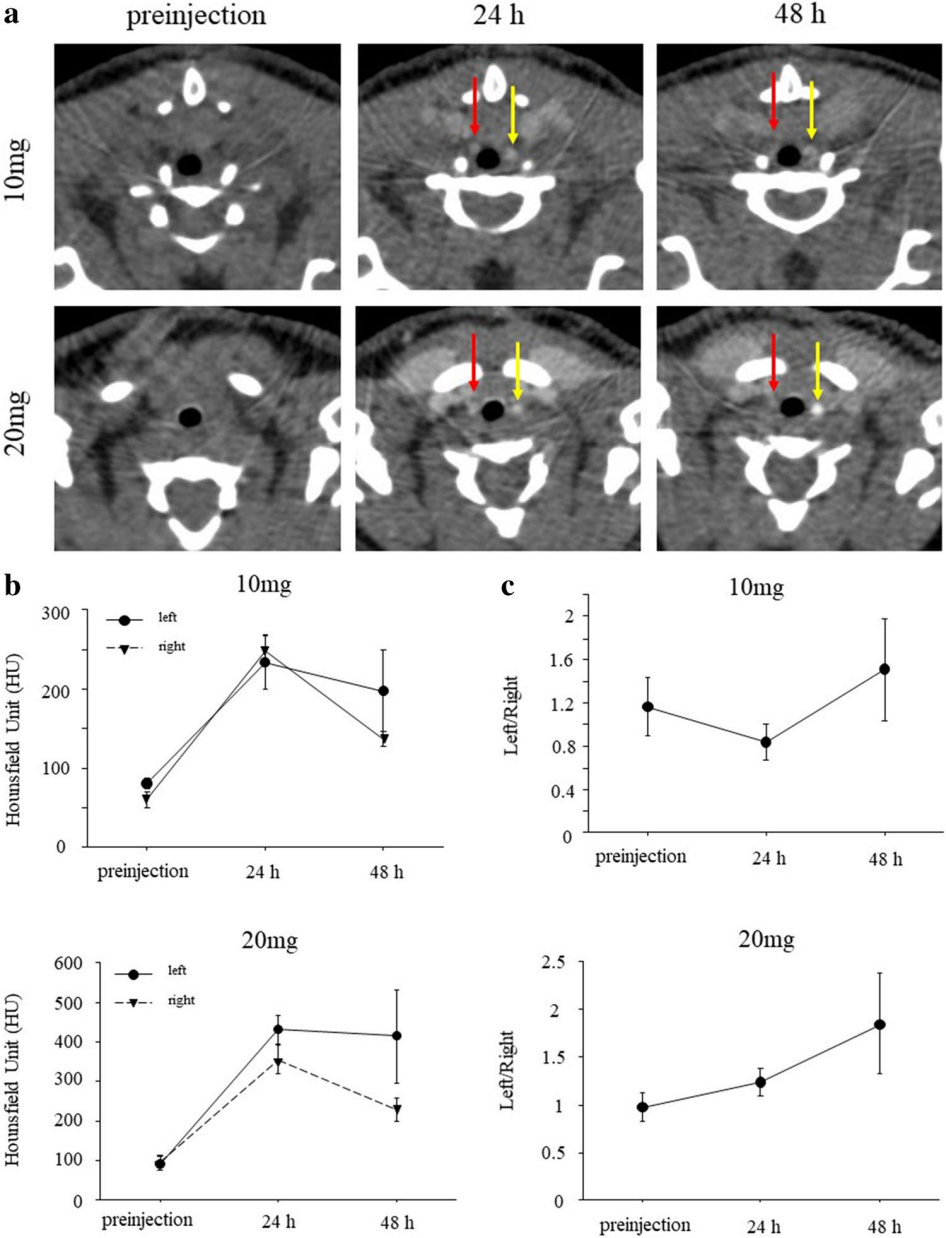
*In vivo* CT imaging of murine carotid arteries after injection of GNPs. **a** Representative serial *in vivo* CT images before and after injection of GNPs at a dose of 10 mg/mouse (top) or 20 mg/mouse (bottom). CT images exhibited contrast enhancement in ligated left (yellow arrows) and non-ligated right (red arrows) carotid arteries at 24 and 48 h after injection of GNPs. **b** The CT attenuation values showed no significant differences between ligated left carotid arteries and non-ligated right carotid arteries in both 10 mg and 20 mg groups. 10 mg, *p* = 0.4417; 20 mg, *p* = 0.2519. **c** The ratio of the CT attenuation values of ligated left carotid arteries to non-ligated right carotid arteries at 48 h was higher than that at 24 h in both 10 mg and 20 mg groups, although statistical differences were not seen. 10 mg, *p* = 0.285; 20 mg, *p* = 0.1088.

In carotid ligation models, *in vivo* CT images exhibited contrast enhancement in the ligated left and non-ligated right carotid arteries at 24 h and 48 h after injection of GNPs in both the 10 mg and 20 mg groups (Fig. 4a). The serial CT attenuation values showed no significant differences between ligated left carotid arteries and non-ligated right carotid arteries in both groups (10 mg, *p* = 0.4417; 20 mg, *p* = 0.2519; Fig. 4b). The ratio of the CT attenuation values of ligated left carotid arteries to those of non-ligated right carotid arteries was compared between 24 and 48 h (Fig. 4c). The ratio was higher at 48 h than 24 h in both the 10 mg and 20 mg groups, although statistical significance was not observed in either group (10 mg: 0.841 ± 0.165 *vs*. 1.510 ± 0.470, *p* = 0.285; 20 mg: 1.238 ± 0.147 *vs*. 1.853 ± 0.526, *p* = 0.1088).

**Fig. 5:**
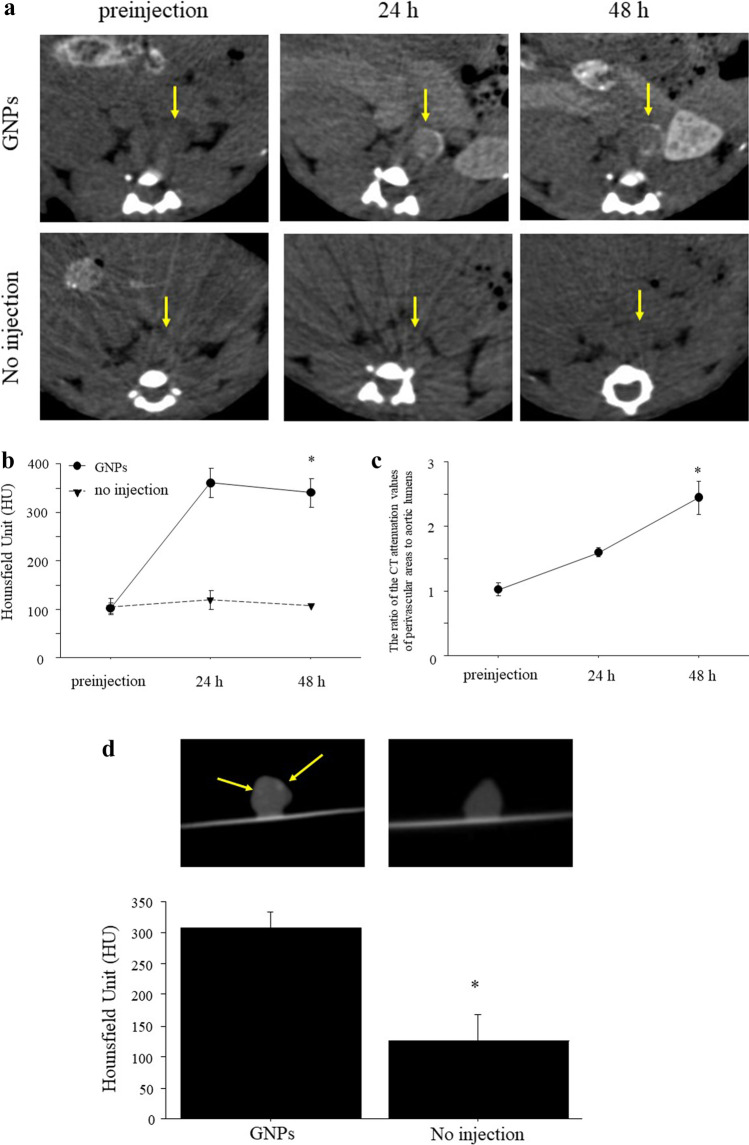
*In vivo* CT imaging of murine AAAs after injection of GNPs. **a** Representative serial *in vivo* CT images of AAAs injected with (top) and without (bottom) GNPs. The CT images exhibited contrast enhancement in the perivascular area (yellow arrows) in AAAs after injection of GNPs (10 mg/mouse) and limited enhancement without injection of GNPs. **b** The CT attenuation values of perivascular area with GNPs were significantly higher than those without GNPs over time. *p = 0.0001. **c** The ratio of the CT attenuation values of perivascular areas to aortic lumens was significantly higher at 48 h than at 24 h in AAAs with GNPs. *p < 0.05. **d** The CT imaging of representative *ex vivo* aorta with and without injection of GNPs (top). Yellow arrows show the accumulation of GNPs in perivascular area. The CT attenuation values of perivascular area in mice with injection of GNPs were significantly higher than those in mice without injection of GNPs (bottom). *p < 0.03.

In AAA models, CT images exhibited contrast enhancement in the perivascular area in AAAs after injection of GNPs, whereas no enhancement was observed without injection of GNPs (Fig. 5a). The serial CT attenuation values of the perivascular area after injection of GNPs were significantly higher than those without injection (*p* = 0.0001, Fig. 5b). The ratio of the CT attenuation values of perivascular areas to those of aortic lumens at 48 h after injection was significantly higher than that at 24 h in AAAs (2.448 ± 0.263 *vs*. 1.595 ± 0.071, *p* < 0.05; Fig. 5c). *Ex vivo* imaging showed that the CT attenuation values of the perivascular area in mice injected with GNPs were significantly higher than those in mice without injection (308.4 ± 24.9 *vs*. 126.3 ± 42.2, *p* < 0.03; Fig. 5d).

## Discussion

In this study, we demonstrated that CT imaging with GNPs can detect vascular inflammation in murine carotid ligation and AAA models. In addition, *in vitro* laser exposure reduced the viability of the macrophages incubated with GNPs.

Imaging techniques for vascular inflammation may be useful for the characterization of biological activity in atherosclerosis and aneurysm. Molecular imaging of vascular inflammation is a promising approach to improve the detection, understanding, and characterization of the progress of atherosclerosis and aneurysm noninvasively [3, 4, 23, 24]. Atherosclerotic lesions and AAAs often contain abundant macrophages, which play crucial roles not only in the development of atherosclerosis and AAAs, but also in the disruption of vulnerable atherosclerotic plaques and AAAs. Therefore, macrophages have emerged as key imaging and therapeutic targets for atherosclerosis and AAAs.

X-ray CT imaging is particularly useful as a diagnostic tool for various diseases in terms of resolution, efficiency, and cost [13, 15]. Iodine contrast agents are widely used for CT imaging because of their high X-ray absorption coefficients. However, they have several limitations including short circulation, allergic reactions, and renal toxicity [13]. Nanoparticle-based contrast agents with long circulation and good biocompatibility may enable safe and reliable noninvasive imaging [25, 26]. Previous studies have shown that GNPs are promising materials for CT contrast agents because of their prolonged blood circulation, good biocompatibility, and higher attenuation coefficient compared to the iodine contrast agents [13–16].

GNPs can be used for visualization of the vascular network and kidneys, as well as tumors and areas of inflammation [20, 27]. Factors such as size, shape, surface charge, and surface functionalization affect the cellular uptake of nanoparticles. Chhour et al. [28] reported that 50 nm and 75 nm GNPs functionalized poly(ethylene-glycol) carboxylic acid ligands (PCOOH) showed higher cellular uptake than 15 nm and 25 nm GNPs functionalized PCOOH. On the other hand, GNPs functionalized carboxylic acid between 15 and 50 nm exhibited high cellular uptake. Because 15-nm GNPs showed a higher contrast than 1.9 nm GNPs in both *in vitro* and *in vivo* [10], 15-nm GNPs were used in this study. The surface functionalization of nanoparticles plays a key role in toxicity reduction. Most GNPs show no toxicity *in vitro* [28, 29]. In addition, GNPs mainly accumulate in the liver and spleen without inducing organ toxicity after intravenous administration in mice [30, 31]. In this study, we showed that the viability of macrophages incubated with GNPs at Au concentrations from 50 to 200 μg/ml was the same as that of macrophages incubated without GNPs (Fig. 2), suggesting that GNPs are nontoxic in the given concentration range and available for *in vivo* imaging. Then, we have demonstrated *in vivo* CT imaging of vascular inflammation with GNPs in murine carotid ligation and AAA models. The CT images exhibited contrast enhancement of atherosclerotic lesions and aortic aneurysms after injection of GNPs (Figs. 4a and 5a), suggesting the accumulation of GNPs at inflammatory sites. The ratio of the CT attenuation values of inflammatory sites to normal sites was higher at 48 h than at 24 h after injection in AAA models due to prolonged blood circulation of GNPs (Fig. 5c). Although *in vivo* imaging at a short time point (24 h) could detect the inflammatory sites, serial imaging over a longer period may be useful to further improve contrast enhancement. The ratio of the CT attenuation values of ligated left carotid arteries to those of non-ligated right carotid arteries between 24 and 48 h in carotid ligation models showed no significant difference (Fig. 4c), which may be due to the small area of inflammatory sites in ligated carotid arteries compared to abdominal aortas. The number of GNPs estimated by the CT attenuation values is close to one in a previous study [10]. However, quantitative data are useful to evaluate the accumulation of GNPs *in vitro* and *in vivo*.

Another potential application of GNPs is photothermal therapy. GNPs, including gold nanorods, gold nanoshells, and gold nanocages, exhibit optical absorption in the NIR region [18–20]. These nanoparticles showed therapeutic efficacy for solid tumors in animal models using continuous-wave NIR laser light irradiation. A pulsed laser can reach a higher temperature and penetrate more deeply into the tissue than a continuous-wave laser [32, 33]. Therefore, we used a pulsed laser to perform an *in vitro* laser irradiation study and demonstrated a significant reduction in viability in macrophages incubated with GNPs by pulsed NIR light irradiation (Fig. 3). A different setting of laser intensity was used in each concentration group. An optical parametric oscillator equipped in the laser system was able to change the output wavelength. However, it was difficult to stabilize the laser intensity due to the angle of nonlinear optical crystals and temperature. Thus, comparing cell viability results between 100 and 200 μg/ml may be challenging. Ghaghada et al. [34] recently reported the detection of aortic degeneration in a murine aortic aneurysm model using gold nanoparticles. They performed CT angiography (CTA) and delayed CT imaging (CTD) for various stages of aortic aneurysm and dissection and showed that CTD enabled the detection of aortic injury in not only moderate-advanced stages but also early stages. Although the detection of AAAs was seen only in the advanced stage in this study, we showed the detection of carotid atherosclerosis and the potential for therapy for vascular inflammation using NIR laser. However, reduced cellular viability was achieved only by high-intensity laser irradiation and long irradiation time, and the mechanism of reduced viability was unclear. Because the blood flow through blood vessels leads to insufficient heating of the target [35], it may be difficult to induce the reduction of *in vivo* cell viability under the same laser conditions as *in vitro*. Further studies are required to examine the effects and setting of laser irradiation.

## Conclusions

In conclusion, *in vivo* CT imaging with GNPs successfully depicted experimental carotid atherosclerosis and AAAs, and NIR laser irradiation reduced the viability of macrophages incubated with GNPs. Thus, GNPs have a strong potential for noninvasive CT imaging and therapy for vascular inflammation.
